# Senescent T Cell Induces Brown Adipose Tissue “Whitening” *Via* Secreting IFN-γ

**DOI:** 10.3389/fcell.2021.637424

**Published:** 2021-03-04

**Authors:** Xiao-Xi Pan, Kang-Li Yao, Yong-Feng Yang, Qian Ge, Run Zhang, Ping-Jin Gao, Cheng-Chao Ruan, Fang Wu

**Affiliations:** ^1^Department of Cardiovascular Medicine, State Key Laboratory of Medical Genomics, Shanghai Key Laboratory of Hypertension, Department of Hypertension, Ruijin Hospital and Shanghai Institute of Hypertension, Shanghai Jiao Tong University School of Medicine, Shanghai, China; ^2^Department of Geriatrics, Ruijin Hospital, Shanghai Jiao Tong University School of Medicine, Shanghai, China; ^3^Department of Physiology and Pathophysiology, School of Basic Medical Sciences, Shanghai Key Laboratory of Bioactive Small Molecules, Fudan University, Shanghai, China

**Keywords:** senescence, T cell, adipose tissue, preadipocyte, brown adipocyte differentiation, IFN-γ

## Abstract

Aging-associated chronic inflammation is a key contributing factor to a cluster of chronic metabolic disorders, such as cardiovascular disease, obesity, and type 2 diabetes. Immune cells particularly T cells accumulate in adipose tissue with advancing age, and there exists a cross talk between T cell and preadipocyte, contributing to age-related adipose tissue remodeling. Here, we compared the difference in morphology and function of adipose tissue between young (3-month-old) and old (18-month-old) mice and showed the phenomenon of brown adipose tissue (BAT) “whitening” in old mice. Flow cytometry analysis suggested an increased proportion of T cells in BAT of old mice comparing with the young and exhibited senescent characteristics. We take advantage of coculture system to demonstrate directly that senescent T cells inhibited brown adipocyte differentiation of preadipocytes in adipose tissue. Mechanistically, both *in vitro* and *in vivo* studies suggested that senescent T cells produced and released a higher level of IFN-γ, which plays a critical role in inhibition of preadipocyte-to-brown adipocyte differentiation. Taken together, the data indicate that senescent T cell-derived IFN-γ is a key regulator in brown adipocyte differentiation.

## Introduction

Advancing age is a major risk factor for chronic metabolic diseases, including cardiovascular disease and type 2 diabetes (Partridge et al., 2018). It has become evident that adipose tissue plays an endocrine function, not merely an energy reservoir pool, and exerts a fundamental influence on metabolic regulation (Oikonomou and Antoniades, 2019; Scheja and Heeren, 2019). Adipose tissue is classified as white adipose tissue (WAT) and brown adipose tissue (BAT). BAT has been considered a key for thermogenesis to maintaining body temperature, while WAT stores and releases lipids and is involved in promoting inflammation (Bartelt and Heeren, 2014; van Eenige et al., 2018). Extensive studies have indicated that WAT could obtain multilocular lipid droplet morphology and expression of BAT-specific genes such as Ucp1, Pparg, and Pgc1a in response to various stimuli (Harms and Seale, 2013). In the contrast, BAT “whitening” refers to acquisition of white adipocyte characteristics with enlarged lipid droplets and loss of normal structure and function of brown adipocyte (Shimizu et al., 2014). Age-related alteration in adipose tissues is manifested on the distribution and composition, as well as a decline in adipose tissue quality and function.

Adipose tissue is composed mainly of adipocytes that interact with other non-adipocyte cells including pre-adipocytes or progenitor cells, endothelial cells, macrophages, and lymphocytes, all of which perform a series of functions for adipose tissue homeostasis (Hafidi et al., 2019). Aging is closely associated with a low-grade systemic inflammation, and innate and adaptive immune systems are influenced by aging. Macrophages, T cells, and other immune cells accumulate with advancing age. Among these, T cells are most susceptible to the detrimental effects of aging and tend to polarize to a proinflammatory phenotype, producing high level of IFN-γ, tumor necrosis factor α (TNF-α), IL-6, and so on (Zanni et al., 2003; Garg et al., 2014; Kalathookunnel Antony et al., 2018). Immune cells infiltrated in adipose tissue have been implicated as either positive or negative regulators in adipogenesis (Brestoff et al., 2015; Lee et al., 2015; Wang and Seale, 2016). Emerging evidence suggests that T cells might participate in regulating differentiation of pre-adipocytes (Wu et al., 2007; Park et al., 2019). However, little attention has been given to contribution of senescent T cells to adipogenic differentiation.

In the present study, we investigated whether T cell senescence in adipose tissue contributed to adipose tissue dysfunction, resulting in age-related metabolic disorders. We verified the BAT whitening in old mice accompanied with increased proportion of T cells. We provided the direct evidence that senescent T cells inhibited the brown adipocyte differentiation of preadipocytes, and white adipocytes formation promoted T cell secreting inflammatory cytokines in turn. Impressively, the inhibitory effect of senescent T cells on BAT whitening was attenuated by blocking IFN-γ. Overall, our findings suggested that senescent T lymphocytes play a negative role in regulation of brown adipocyte formation as well as inflammatory response of adipose tissue *via* secreting inflammatory cytokines, particularly IFN-γ.

## Materials and Methods

### Animals

Wild-type (WT) male mice including young (3-month-old) and old (18-month old) mice, as well as RAG1−/− mice (t004753) were obtained from Model Animal Research Center of Nanjing University (GemPharmatech, Jiangsu, China). All mice were on C57BL/6J background. All animal experiments were approved by institutional guidelines established by the Committee of Ethics on Animal Experiments of Shanghai Jiao Tong University School of Medicine.

### Histological Analysis

In order to perform hematoxylin-eosin (H&E) staining, paraformaldehyde-fixed adipose tissues were embedded in paraffin and then cut into 5-μm transverse sections. For immunofluorescence staining, sections were boiled in sodium citrate buffer to retrieve antigen and blocked with 5% bovine serum albumin. Then the sections were then incubated with primary antibodies at 4°C overnight, and incubated with the respective fluorochrome-conjugated secondary antibodies, followed by nuclear staining with 4,6-diamidino-2-phenylindole (DAPI). Images were observed with the Carl Zeiss Axio Imager M2 microscope (Carl Zeiss Corporation, Germany).

### Flow Cytometry Analysis

Adipose tissues including interscapular brown adipose tissue (BAT), subcutaneous adipose tissue (SAT), or epididymal visceral adipose tissue (VAT) were isolated from young or old WT mice and cut into small pieces, which digested with collagenase solution (2 mg/ml collagenase type II in RPMI 1,640 medium) at 37°C for 30 min. The samples were filtered with 40 μM strainers and centrifuged at 450 × *g* for 5 min at 4°C. Samples were analyzed for specific surface and intracellular markers by Beckman CytoFLEX (Beckman Coulter, United States). Antibodies used for staining were as follows: Fixable Viability Dye-APC-eFlour780 (65-0865-14, eBioscience), CD45-Violet 510 (103138, BioLegend), CD3e-Violet 605 (100351, BioLegend), IFN-γ-APC (17-7311-81, eBioscience), CD28-PE (12-0281-82, eBioscience), CD44-APC-eFlour780 (47-0441-82, eBioscience), and CD62L-eFlour450 (48-0621-80, eBioscience).

### Cell Culture and Differentiation

Stromal vascular fraction (SVF) cells isolated from BAT or SAT of WT mice were maintained in pre-adipocyte medium (DMEM/F12, 10% FBS and 1% penicillin/streptomycin) at 37°C with 5% CO_2_. SVF cells at 80% confluences were transferred into brown adipocyte (BA) differentiation medium or white adipocyte (WA) differentiation medium and extended for 8 days until adipocyte formation as previously described (Pan et al., 2019). Splenic CD3^+^ T cells isolated by negative selection kit (8804-6820-74, eBioscience) from young or old mice were cultured in complete medium (RPMI1640 with 10% FBS and 1% penicillin–streptomycin) and activated by anti-CD3 antibody (2 μg/ml) (16-0031-85, eBioscience) and anti-CD28 antibody (1 μg/ml) (16-0281-85; eBioscience).

To investigate whether T cells could regulate adipogenic differentiation of SVF cells, SVF cells were cocultured with CD3^+^ T cells of young and old mice, respectively. After coculture for 8 days, T cells and differentiated SVF cells were collected, respectively, for further analysis. To block IFN-γ, IFN-γ-neutralizing antibody (10 μg/ml) was added to cultured media at the beginning of cocultivation.

### Western Blotting Analysis

Total protein was extracted from adipose tissues or cultured cells, using RIPA lysis buffer with protease inhibitors cocktail (Selleck). Protein lysates were resolved by SDS-PAGE and transferred onto PVDF membranes. Membranes were then incubated with primary antibodies, including anti-UCP-1 (Abcam, ab23841), anti-perilipin (Abcam, ab61682), anti-APN (R&D, AF1119), anti-PPARγ (Santa Cruz, sc-7273), anti-PGC1α (Santa Cruz, sc-13067), and β-actin for normalization. Subsequently, incubation with HRP-conjugated antibodies, and then protein signals were detected with electrochemiluminescence (ECL) detection reagents.

### Quantitative Real-Time Polymerase Chain Reaction

Total RNA from adipose tissues or cultured cells was extracted with Trizol (Invitrogen) followed by reverse transcription with 1 μg total RNA according to the manufacturer’s protocol. Quantitative real-time polymerase chain reaction (qRT–PCR) were performed with SYBR qPCR Master Mix (Vazyme, China) on ABI PRISM 7900 machine (Applied Biosystems). β-Actin was used as the standard reference. The data were calculated using the 2-ΔΔCT method. Sequences of primers were as follows. mβ-Actin: forward: 5′-CTA AGG CCA ACC GTG AAA AGA T-3′, reverse: 5′-GGG ACA GCA CAG CCT GGA T-3′; Ucp1: forward: 5′-AGG CTT CCA GTA CCA TTA GGT-3′, reverse: 5′-CTG AGT GAG GCA AAG CTG ATT T-3′; Pparg: forward: 5′-TTA GAT GAC AGT GAC TTG GC-3′, reverse: 5′-TCT TCT GGA GCA CCT TGG-3′; Ppargc1a: forward: 5′-GAA GTG GTG TAG CGA CCA ATC-3′, reverse: 5′-AAT GAG GGC AAT CCG TCT TCA-3′; Plin1: forward:5′-GTG CAA TGC CTA TGA GAA GGG TGT AC-3′, reverse: 5′-GTA GAG ATG GTG CCC TTC AGT TCA GA-3′; Adipoq: forward: 5′-GAT GGC AGA GAT GGC ACT CC-3′, reverse: 5′-CTT GCC AGT GCT GCC GTC AT-3′; Ifng: forward: 5′-ATC TGG AGG AAC TGG CAA AA-3′, reverse: 5′-TTC AAG ACT TCA AAG AGT CTG AGG-3′; Il10: forward: 5′-TGG ACA ACA TAC TGC TAA CCG-3′, reverse: 5′-GGA TCA TTT CCG ATA AGG CT-3′; Il6: forward: 5′-TGT GCA ATG GCA ATT CTG AT-3′, reverse: 5′-GGT ACT CCA GAA GAC CAG AGG A -3′.

### Statistical Analysis

Results were presented as mean ± SEM and analyzed using Prism (Graph Pad Software). Statistical comparison of data between two groups was tested by unpaired two-tailed Student’s *t* test. The value of *P* < 0.05 was considered to be statistically significant.

## Results

### Aging Induces Brown Adipose Tissue “Whitening” in Adipose Tissue in the Whole Body

**FIGURE 1:**
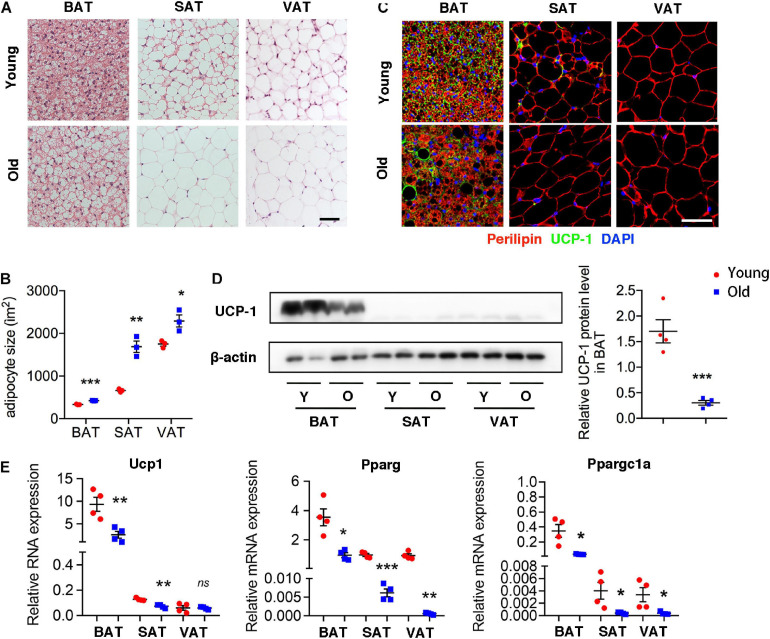
Aging induced brown adipose tissue whitening. **(A)** Representative H&E-stained sections of BAT, SAT, and VAT of young and old mice. **(B)** The quantitative analysis of adipocyte size. **(C)** Representative immunofluorescence of perilipin and UCP-1 in adipose tissues from young and old mice. 4′,6-Diamidino-2-phenylindole (DAPI) was used to detect nucleus. **(D)** Representative Western blot for UCP-1 expression in adipose tissues and their quantitation. **(E)** qRT-PCR analysis of mRNA expression levels of Ucp1, Pparg, and Ppargc1a in adipose tissues. Scale bar, 50 μm. *P < 0.05; **P < 0.01; ***P < 0.001 vs. young mice, *n* = 4 per group.

H&E staining of different adipose tissue showed dramatically differences in size and characterization. The size of adipocytes in old mice was significantly larger than those in young mice, including BAT, SAT, and VAT. Furthermore, brown adipocytes contained multilocular lipid droplets in BAT of young mice, while adipocytes in aged mice tended to have larger locular lipid droplets, a morphology more similar to a white adipocyte relative to those in young mice (Figures 1A,B). More importantly, UCP-1 expression fundamentally decreased in BAT of old mice comparing with that in young mice. Moreover, UCP-1 was weakly detected in the SAT of young mice, whereas it was not detected in old mice (Figure 1C). Western blot and qRT-PCR analysis also demonstrated that a lower level of UCP-1 both in BAT of old mice than that in young mice (Figures 1D,E). qRT-PCR analysis further indicated that genes involved in brown adipocyte formation, such as Pparg and Ppargc1a decreased in these adipose tissues of old mice. All of those indicated that aging inhibited brown adipocyte formation, resulting in BAT whitening.

### Infiltration of Senescent T Cell in Adipose Tissue of Old Mice

**FIGURE 2:**
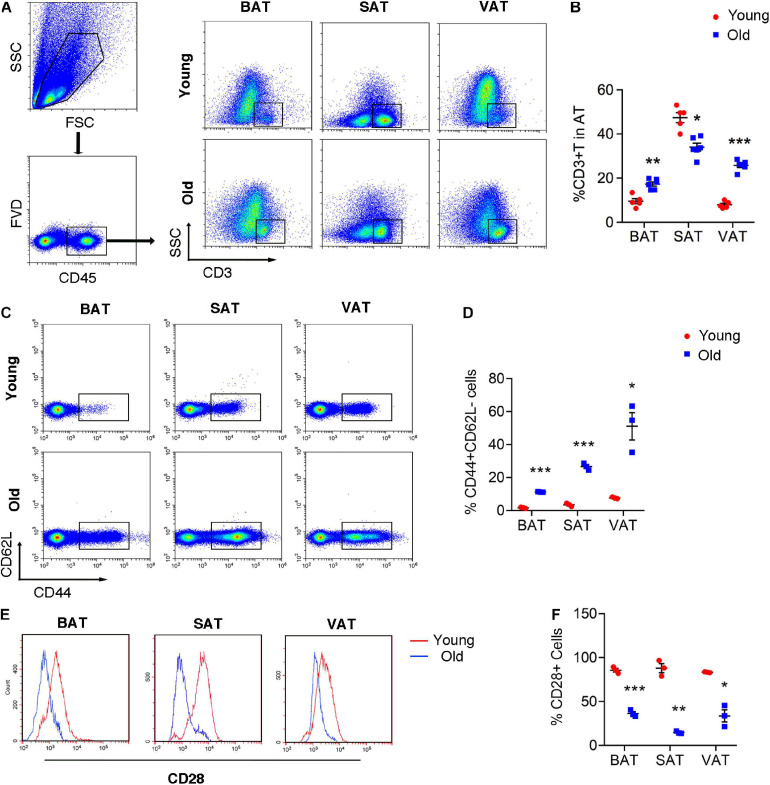
T cell senescence in various adipose tissues of old mice. **(A,B)** Flow cytometric analysis of CD45^+^CD3^+^ T cells in AT of young and old mice and their quantitation. **(C,D)** Representative flow cytometric analysis of CD44 subsets in T cells isolated from adipose tissue and their quantification. **(E,F)** Representative flow cytometry of CD28^+^ T cells in adipose tissue from young or old mice and their quantification. *P < 0.05; **P < 0.01; ***P < 0.001 vs. young mice, *n* = 3∼6 per group.

Given the emerging evidence of immune cells involved in the adipocyte thermogenesis, we firstly detected T cell infiltration in the adipose tissue during aging. Flow cytometric analysis revealed the two- to threefold amount of T cells in BAT and VAT of old mice than those in young mice. However, the proportion of T cells decreased in SAT of old mice (Figures 2A,B). More importantly, T cell isolated from adipose tissue of old mice, regardless of BAT, SAT, or VAT, exhibiting senescent features, including loss of CD28 and expansion of CD44^+^CD62L^–^ memory T cells (Figures 2C–F).

**FIGURE 3:**
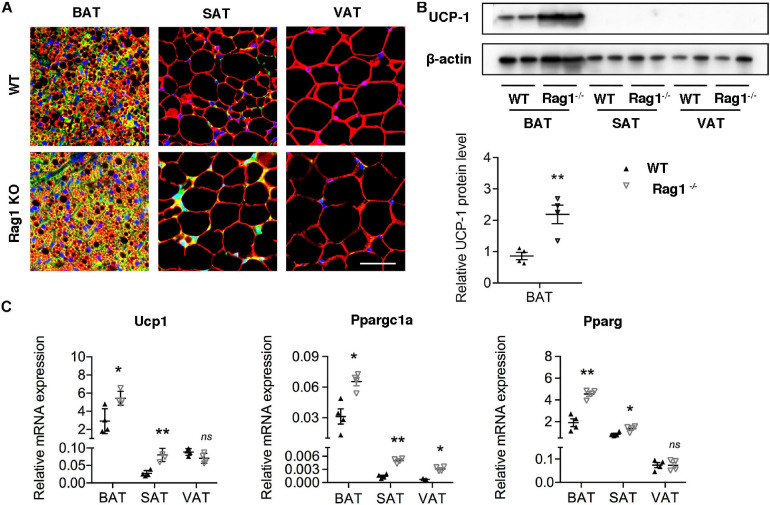
T cell deficiency promoted brown adipocyte formation. **(A)** Representative immunofluorescence of perilipin (red) and UCP-1 (green) in adipose tissues from WT and RAG1 KO mice. **(B)** Representative Western blot and quantitation for UCP-1 in BAT, SAT, and VAT of WT and Rag1 KO mice. **(C)** qRT-PCR analysis of relative mRNA expression of Ucp1, Pparg, and Ppargc1a in AT of WT and Rag1 KO mice. *P < 0.05; **P < 0.01 vs. WT mice, *n* = 4 per group.

Considering the probable function of T cells in regulation of brown adipocyte formation, we assessed brown adipocyte-associated protein and mRNA expression in adipose tissues at different location in age- and weight-matched WT and Rag1 KO male mice that lack T and B cells. UCP-1 expression of both protein and mRNA were found to be higher in BAT of Rag1 KO mice, which was suggestive of an increased proportion of brown adipocytes (Figures 3A–C). Consistent with this, gene expression profiling revealed a higher level of Ppargc1a and Pparg, both of which are critical for brown adipocyte differentiation, indicating that T cells may participate in regulation of brown adipocyte differentiation or adipocyte browning (Figure 3C).

### Senescent T Cell Inhibits Brown Adipocytes Differentiation of SVF Cells

**FIGURE 4:**
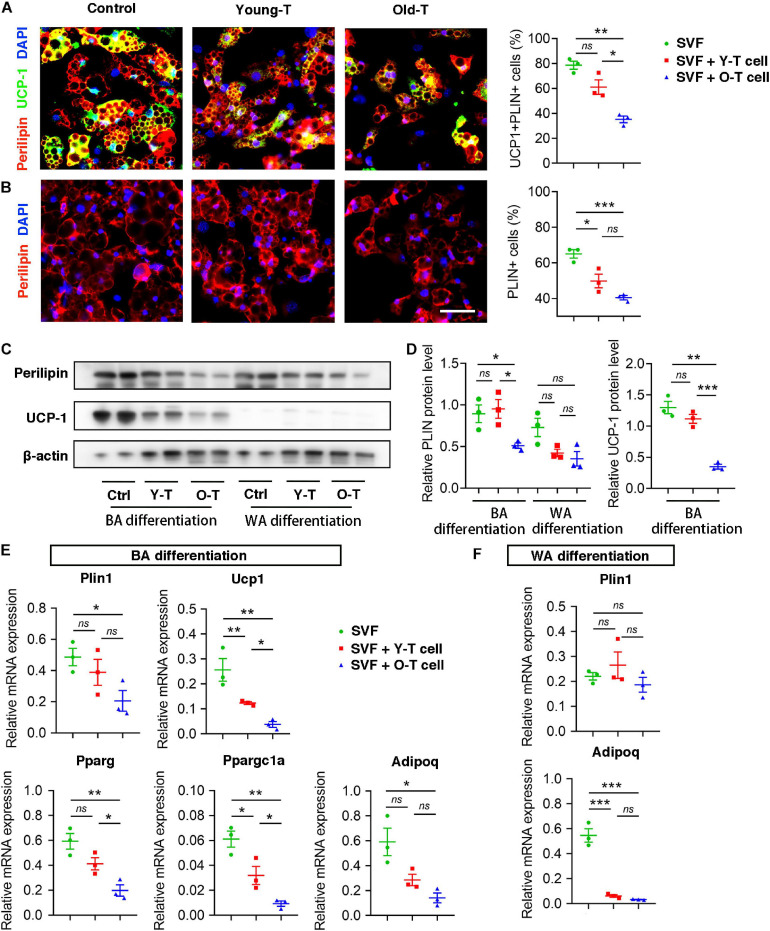
Senescent T cells inhibited brown adipogenic differentiation of SVF cells. **(A,B)** The adipogenic differentiation was determined by costaining of perilipin and UCP-1, and their quantitative analysis. Scale bar, 50 μm. **(C,D)** Representative western blot and quantitation for relative UCP-1 and perilipin (PLIN) protein levels. **(E,F)** qRT-PCR analysis of relative mRNA expression of Plin1, Ucp1, Pparg, and Ppargc1a and Adipoq in SVF cells under BA differentiation **(E)** and WA differentiation **(F)**. *P < 0.05; **P < 0.01; ***P < 0.001, *n* = 3 independent experiments.

To further investigate the role of senescent T cells in preadipocyte-to-brown adipocyte differentiation, SVF cells from BAT were cocultured with young or senescent T cells, respectively, through Transwell system. In addition, these SVF cells were differentiated toward brown adipocytes or white adipocytes, respectively. Interestingly, SVF cells cocultured with senescent T cells exhibited a significant decrease of brown adipocyte formation comparing with that with young T cells. Both young and senescent T cells seem to inhibit preadipocyte-to-white adipocyte differentiation, but there was no significant difference between two groups (Figures 4A,B). This trend was further confirmed by the protein levels of UCP-1 as well as mRNA expression. Quantitatively, old T cell dramatically suppressed expression of UCP-1 and perilipin protein in SVF cells (Figures 4C,D). Likewise, qRT-PCR data showed that senescent T cells inhibited expression of genes involved in brown adipogenic differentiation, including Ucp1, Pparg, and Ppargc1a. In addition, Plin1 and Adipoq were decreased in SVF with senescent T cells (Figures 4E,F). In line with BAT-preadipocyte differentiation, senescent T cells impaired SAT preadipocytes to differentiate toward brown adipocytes and white adipocytes (Supplementary Figure 1).

### Senescent T Cell-Derived IFN-γ Involved in Regulation of Brown Adipogenic Differentiation

**FIGURE 5:**
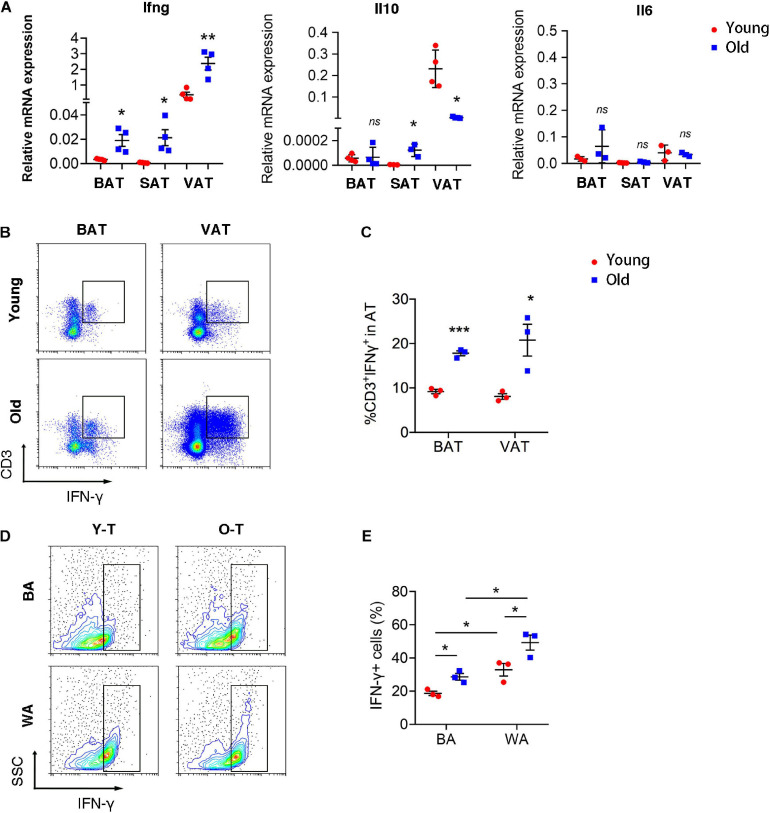
T cell-derived IFN-γ was increased in adipose tissues of old mice. **(A)** qRT-PCR analysis of mRNA expression level of Ifng, Il10, and Il6 in adipose tissue. **(B,C)** Flow cytometric analysis of CD3^+^IFN-γ^+^ in BAT and VAT of young and old mice. *P < 0.05; **P < 0.01; ***P < 0.001 vs. young mice, *n* = 3 per group. **(D,E)** The production of IFN-γ in T cells cocultured with SVF cells under adipogenic differentiation. *P < 0.05, *n* = 3 independent experiments.

To explore the underlying mechanism that senescent T cells inhibit pre-adipocyte-to-brown adipocyte differentiation, our previous studies had demonstrated that senescent T cells secret a higher level of IFN-γ than young T cells (Pan et al., 2020). Thus, we detected inflammatory gene expression, including Ifng, Il10, and Il6 in varying adipose tissues of young and old mice. Expression of Ifng was significantly increased in BAT, SAT, as well as VAT of old mice, compared with adipose tissue of young mice. However, Il10 expression was enhanced in SAT whereas being decreased in VAT of old mice compared with the young ones. In addition, there were no significant differences in Il6 expression between young and old mice (Figure 5A). As shown in Figure 5B, the percentage of IFN-γ^+^ T cells was substantially higher in BAT and VAT of old mice than those in young mice (Figures 5B,C). Notably, both young and senescent T cells that cocultured with SVF cells under white adipocyte differentiation produced a higher level of IFN-γ than cells under brown adipogenic differentiation. Furthermore, senescent T cells secreted more IFN-γ than young T cells (Figures 5D,E).

**FIGURE 6:**
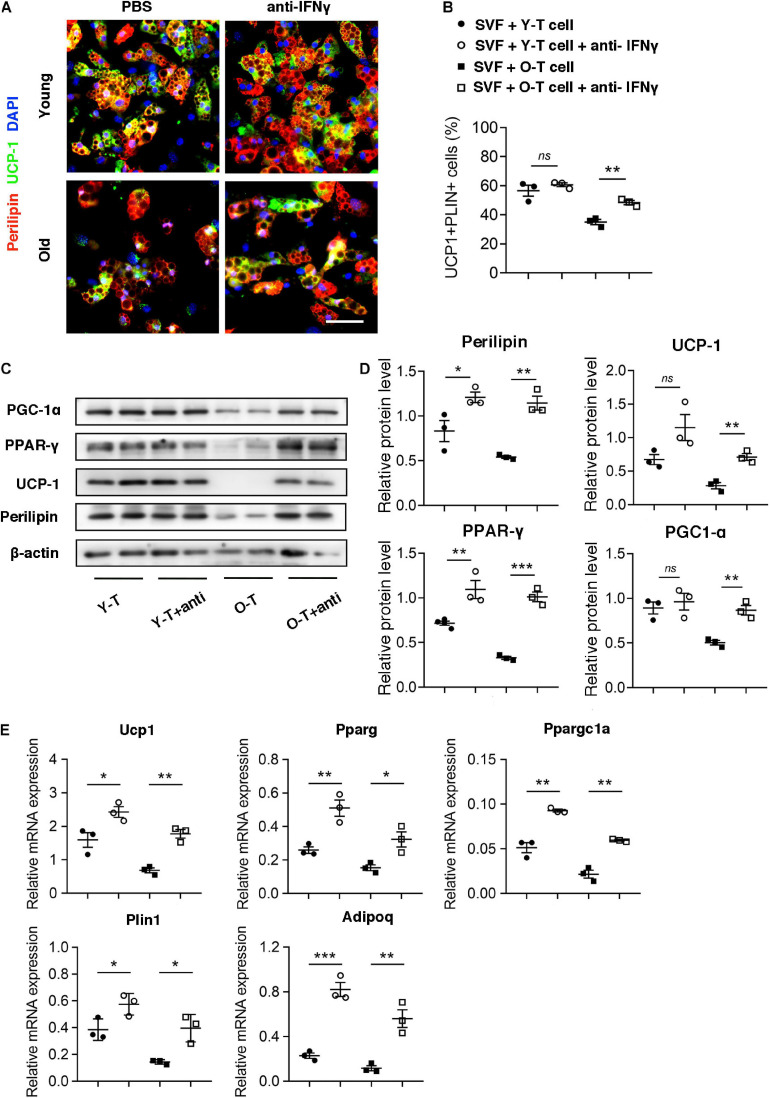
Blockade of IFN-γ rescued the brown adipogenic differentiation potential of SVF cells cocultured with senescent T cells. **(A)** The adipogenic differentiation was determined by costaining of perilipin and UCP-1. Scale bar, 50 μm. **(B)** The quantitative analysis of immunofluorescence. **(C,D)** Representative western blot and their quantitation. **(E)** The adipogenic differentiation was determined by qPCR of specific markers. *P < 0.05; **P < 0.01; ***P < 0.001, *n* = 3 independent experiments.

To identify the potential role for IFN-γ in brown adipogenic differentiation of SVF cells from BAT, we administrated IFN-γ-neutralizing antibody to block the adverse effect of IFN-γ on adipogenic differentiation. Remarkably, blockade of IFN-γ rescued the brown adipogenic differentiation capacity of SVF cells cocultured with senescent T cells (Figures 6A,B). Furthermore, there was no significant difference in preadipocyte-to-brown adipocyte differentiation between SVF cells without the presence of T cells under treatment of IFN-γ-neutralizing antibody or PBS (Supplementary Figure 2). In addition, protein level of perilipin and UCP-1 in SVF cells with senescent T cells significantly increased after treatment of IFN-γ-neutralizing antibody, accompanied by an increase in PGC-1α and PPAR-γ expression (Figures 6C,D). In keeping with this, genes associated with brown adipogenic differentiation, including Ucp1, Pparg, and Ppargc1a were significantly upregulated under anti-IFN-γ treatment both in young and senescence groups (Figure 6E). In addition, brown adipocyte differentiation potential of SAT preadipocyte inhibited by senescent T cell was improved by administration of IFN-γ-neutralizing antibody (Supplementary Figure 3). These provided direct evidence that T cell-derived IFN-γ plays an important role in inhibiting brown adipocyte differentiation during aging.

## Discussion

The increasing population of elderlies combined with obesity, especially expansion of visceral fat mainly composed of white adipocytes results in an alarming rise in cardiometabolic disorders (Martyniak and Masternak, 2017). Adipose tissue remodeling during the aging process is a common phenomenon, and it remains unclear how this maladaptive process is developing. Adipose tissue is composed of several different cellular populations, which varies depending on the location and metabolic status of an organism. Our prior studies has demonstrated that there are distinct cellular components in perivascular stromal cells between young and old mice, and aging influence the potential of stromal cells from perivascular adipose tissue (Pan et al., 2019). Furthermore, adipocyte browning capacity under cold exposure was impaired in the context of aging (Pan et al., 2018). Herein, we further unveil the pivotal role of T cells in age-induced decline of brown adipocyte differentiation.

Adipose tissue is in a constant flux with changing cellular populations, and it plays an important role in the regulation of inflammatory status during aging. Inflammation is believed to closely associate with the metabolic diseases (Hotamisligil, 2006). Mounting evidence emphasizes the importance of immune cells, especially T cells in adipose tissue inflammation (McLaughlin et al., 2014, 2017; Han et al., 2017). Many similarities in chronic inflammation in adipose tissue have been identified between obesity and aging, such as an increase number of CD4^+^ or CD8^+^ T cells and a decreased amount of iNK T cells. Moreover, immune cells tend to have proinflammatory phenotypes (Martyniak and Masternak, 2017; Kalathookunnel Antony et al., 2018). A number of studies have clearly demonstrated that T cells in adipose tissue play a role in obesity-associated inflammation. Winer et al. (2009) indicated that lymphocyte-deficient RAG-1 KO mice developed a greater degree of metabolic disorder after 8 weeks of high fat diet, and CD4^+^ (but not CD8^+^) T cell adoptive transfer to RAG1 KO mice reversed weight gain and insulin resistance. On the other hand, CD8^+^ T cells in obese epididymal adipose tissue were significantly increased and preceded the recruitment and activation of adipose tissue macrophages, which lead to the propagation of adipose inflammation (Nishimura et al., 2009). In addition, prior studies show a preferential increase and accumulation of CD153^+^PD-1^+^CD44^hi^CD4^+^ T cell population in VAT of diet-induced obese mice, which suggests a possible link between visceral adiposity and immune aging (Shirakawa et al., 2016). Given the importance of T cell in diet-induced visceral obesity, it is possible that T cell senescence could influence adipose tissue during the aging process. Our data demonstrate that T cells accumulate in BAT and VAT during aging, but SAT exhibited opposite trends of T cell infiltration. Aging leads to adipose tissue redistribution, including a general increase in visceral fat but a reduction in subcutaneous fat (Ponti et al., 2019), which might result in deposition of collagen instead of immune cells, preadipocyte, and other cells in SAT. Although the amount of T cells is decreased in SAT, T cells isolated from three types of adipose tissue in aged mice present senescent features, such as loss of CD28 and increased proportion of CD44^+^CD62L^–^ effector memory T cells and secrete a higher level of proinflammatory factor such as IFN-γ, which provides a clue that function of T cell rather than quantity of cells exerts a profound influence on adipocyte differentiation.

Both aging and obesity can result in adipose tissue remodeling, along with a decline of its function, predisposing humans to development of metabolic disorder and cardiovascular diseases (Neeland et al., 2018; Marcelin et al., 2019). However, age-induced dysfunction of adipose tissue may be distinct from obese-related malfunction of adipose tissues. In the aging process, redistribution of adipose tissue toward visceral or ectopic sites, such as skeletal muscle and epicardium, has a causal or correlative relation with metabolic and cardiovascular diseases. It has been demonstrated that pre-adipocytes or progenitor cells exist in adipose tissue and can differentiate into brown adipocytes or white adipocytes under a specific condition (Berry et al., 2016; Pyrina et al., 2020). This process is disturbed with senescence. Our data showed that adipocytes in BAT of old mice exhibited a characteristic more similar to white adipocyte, which was termed as BAT “whitening.” Low-grade chronic inflammation persists in adipose tissue of aged population and chronic inflammation-induced adipose tissue dysfunction might attribute to impaired brown adipogenic differentiation. Emerging evidence suggests the novel role for T lymphocytes in regulating brown adipocyte formation *via* secreting cytokines. CD8^+^ T cell deficiency enhanced beige adipogenesis in the SAT of Rag1 KO mice, which is mediated by secreted factors such as IFN-γ (Moysidou et al., 2018). iNK T cells remove hypertrophic and proinflammatory adipocytes and induce differentiation of adipocyte progenitor cells, contributing to maintenance of adipose tissue homeostasis (Park et al., 2019). In the present study, accumulation of senescent T cells in BAT of aged mice might lead to a dysfunction in progenitor cell differentiation and then cause adipocytes taking on the appearance of a white adipocyte rather than a brown adipocyte. *In vitro* experiment further provided direct evidence that senescent T cells inhibited brown adipocyte differentiation of pre-adipocytes from both BAT and SAT. Cytokine within adipose tissue is a major mediator of adipose tissue homeostasis and originates from several cell types including adipocytes, pre-adipocytes, as well as immune cells (Coppack, 2001). It has been reported that M1 macrophages impair adipogenesis of 3T3-L1 preadipocytes *via* producing TNF-α (Nisoli et al., 2000; Cawthorn et al., 2007). In contrast, ILC2-derived IL-13 and eosinophil-derived IL-4 together stimulates the expansion of adipocyte progenitor cells and browning of SAT, leading to increase energy expenditure (Brestoff et al., 2015; Lee et al., 2015). Herein, we demonstrated that T cell-derived IFN-γ acted directly on pre-adipocyte to inhibit its brown adipocyte differentiation *via* downregulation of PPARγ expression that is a prime inducer of adipocyte differentiation. Activation of PPARγ is necessary for brown adipocyte differentiation of preadipocyte, and PGC-1α as coactivator of PPARγ also is a key regulator in brown adipogenesis. Of note, blockade of IFN-γ successfully rescued the brown adipocyte differentiation of preadipocyte cocultured with T cells and upregulated level of PPARγ expression. In addition, IFN-γ-neutralizing antibody had no effect on differentiation of preadipocyte to brown adipocyte without the presence of the T cell. These suggested that IFN-γ is the cause for senescent T cell-induced inhibition on BA differentiation of preadipocytes.

**FIGURE 7:**
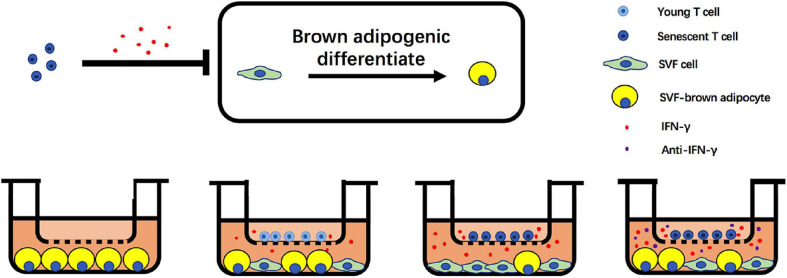
Schematic illustration of adverse effect of T cells on brown adipogenic differentiation of SVF cells regulated by IFN-γ.

In summary, we unveil the critical role of senescent T cell in regulation of aging-induced BAT “whitening.” Notably, an increase of senescent T cell accumulation in BAT of aged mice and a higher level of IFN-γ from senescent T cells inhibit the brown adipogenic differentiation of pre-adipocyte, contributing to adipose tissue remodeling in the elderlies. Notwithstanding the complexity of aging-associated alteration in adipose tissue, these data indicate that targeting T cell-derived IFN-γ might be a potential alternative to treat age-related metabolic disorders (Figure 7).

## Data Availability Statement

The original contributions presented in the study are included in the article/Supplementary Material, further inquiries can be directed to the corresponding authors.

## Ethics Statement

The animal study was reviewed and approved by the Ethics Committee of Animal Experiments of Shanghai Jiao Tong University School of Medicine.

## Author Contributions

C-CR, FW, and X-XP designed the study and wrote the manuscript. K-LY and Y-FY conducted *in vivo* studies. K-LY and RZ performed *in vitro* studies. X-XP and QG performed statistical analyses with assistance from P-JG. All authors contributed to the article and approved the submitted version.

## Conflict of Interest

The authors declare that the research was conducted in the absence of any commercial or financial relationships that could be construed as a potential conflict of interest.
