# Supplementary material for: Senescent T Cell Induces Brown Adipose Tissue “Whitening” Via Secreting IFN-γ
Source: Front Cell Dev Biol. 2021 Mar 4;9:637424. doi: 10.3389/fcell.2021.637424 (PMC7969812; doi:10.3389/fcell.2021.637424)
Supplement: Supplementary file 1 [file Table_1.DOCX]

Supplementary Material

# Supplementary Figures


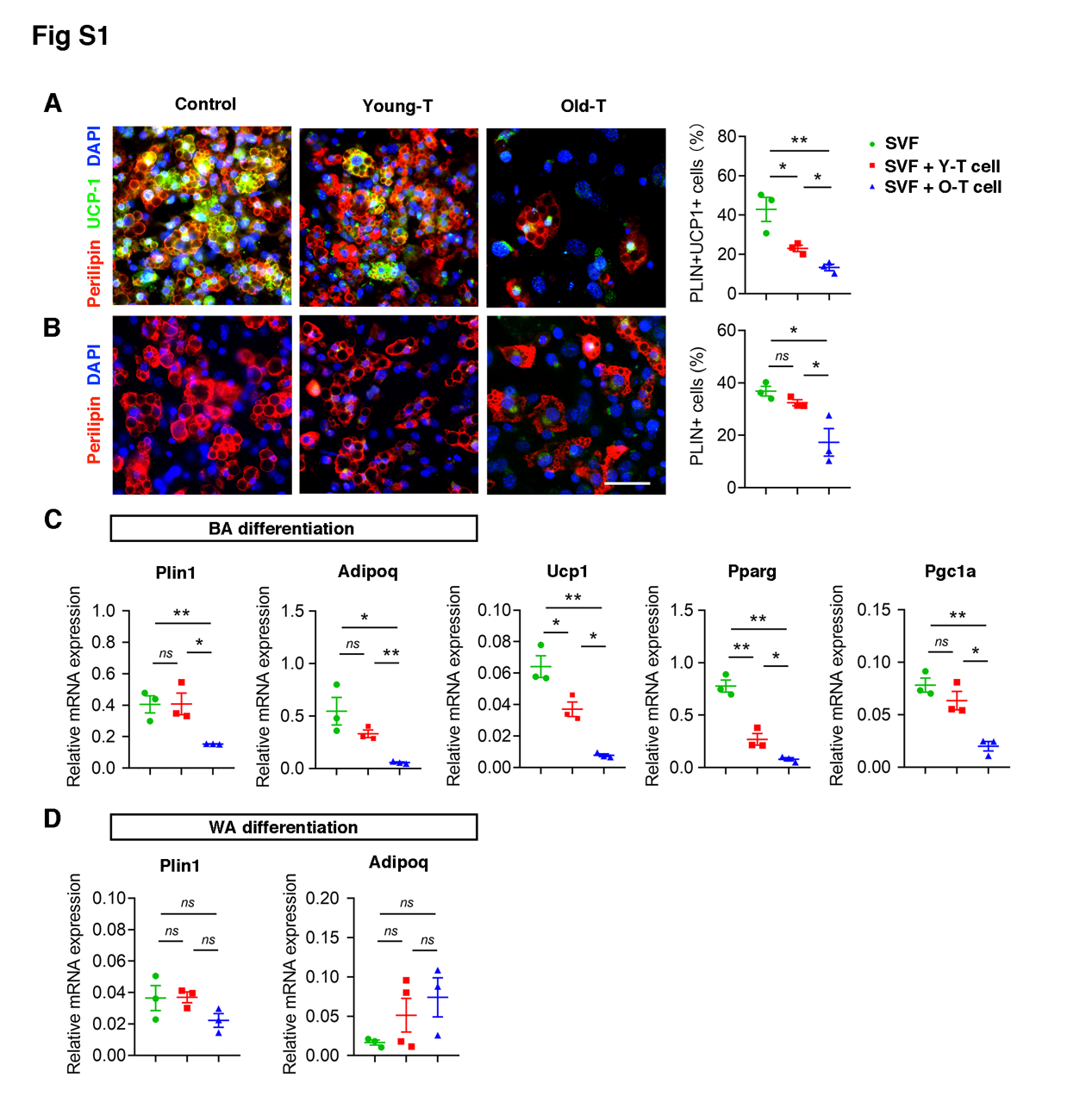


**Supplementary Figure 1.** Senescent T cells inhibited brown adipogenic differentiation of SAT-preadipocyte. A, B) The adipogenic differentiation was determined by co-staining of perilipin (red) and UCP-1 (green), and their quantitative analysis. Scal bar 50 μm. C, D) qRT-PCR analysis of relative mRNA expression of Plin1, Ucp1, Pparg and Ppargc1a and Adipoq in preadipocytes under BA (C) and WA differentiation (D). ∗P < 0.05; ∗∗P < 0.01, n = 3 independent experiments.


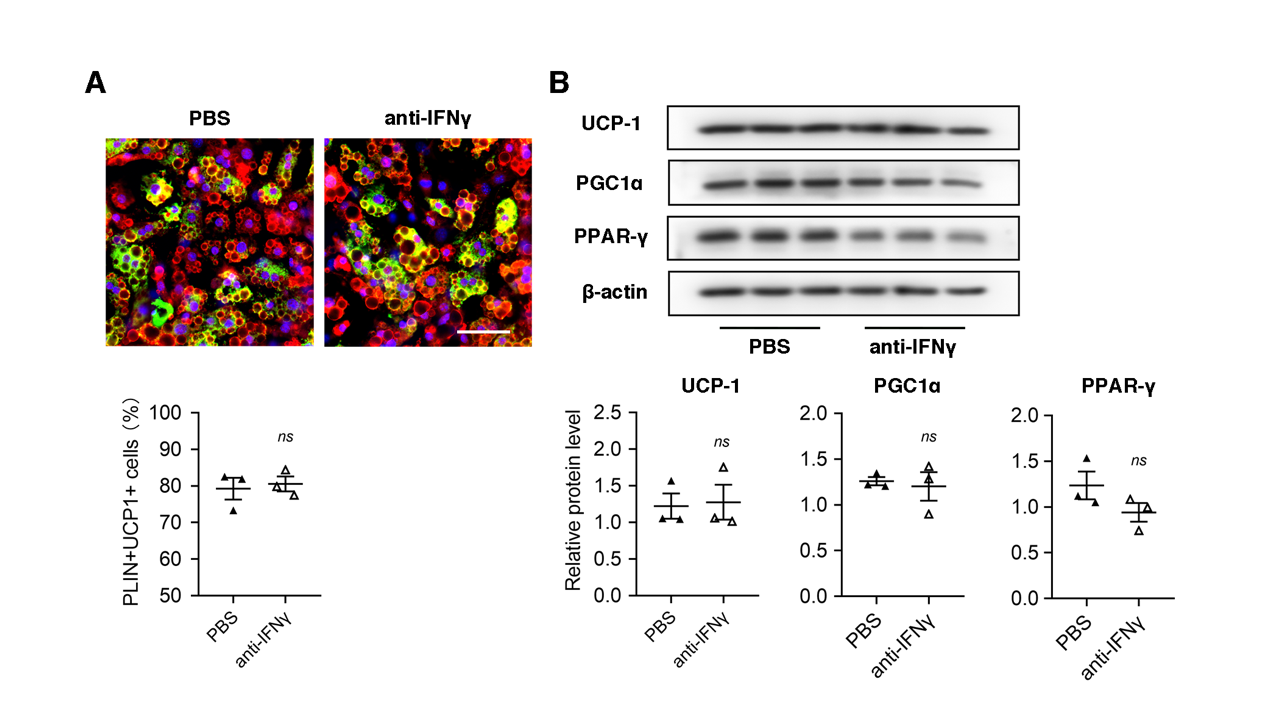


**Supplementary Figure 2.** IFN-γ neutralizing antibody had no effect on regulating of brown adipocyte differentiation. A) The adipogenic differentiation was determined by co-staining of perilipin (red) and UCP-1 (green). The bottom is its quantitative analysis. Scal bar 50 μm. B) Representative western blot and quantitation for relative UCP-1, PGC-1α and PPAR-γ protein levels. n = 3 independent experiments.


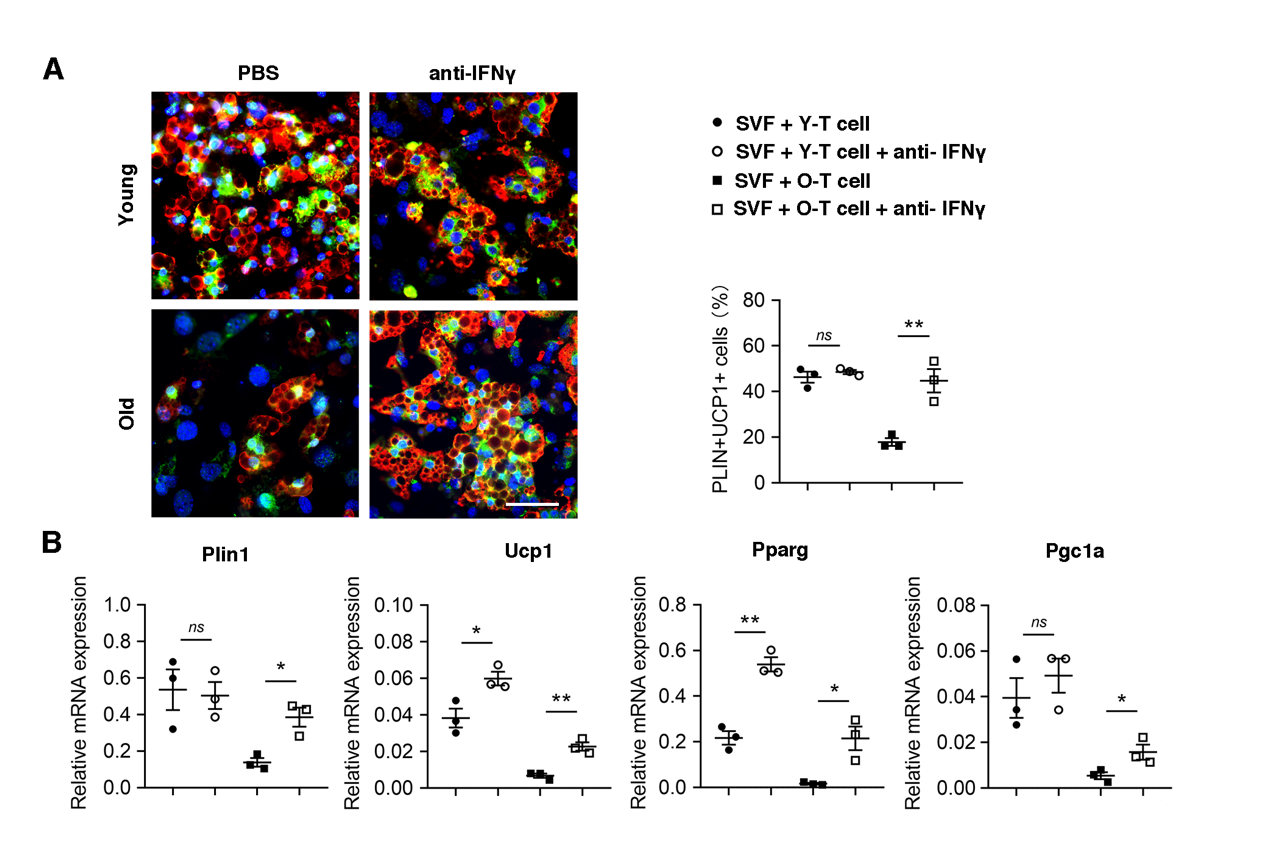


**Supplementary Figure 3.** Blockade of IFN-γ improved the brown adipogenic differentiation potential of SAT-preadipocyte cocultured with senescent T cells. A) The adipogenic differentiation was determined by co-staining of perilipin (red) and UCP-1 (green). And the quantitative analysis of immunofluorescence. Scal bar 50 μm. B) The adipogenic differentiation was determined by qPCR of specific markers. ∗P < 0.05; ∗∗P < 0.01, n = 3 independent experiments.
